# Down-regulation of the klf5-c-Myc interaction due to klf5 phosphorylation mediates resveratrol repressing the caveolin-1 transcription through the PI3K/PKD1/Akt pathway

**DOI:** 10.1371/journal.pone.0189156

**Published:** 2017-12-06

**Authors:** Hui Yang, Qiuxia Chen, Fangyun Sun, Nana Zhao, Lirong Wen, Lin Li, Gai Ran

**Affiliations:** 1 Department of Clinical Nutrition, Kecheng People's Hospital, Quzhou, Zhejiang Province, China; 2 Department of Preventive Medicine, Wenzhou Medical University, Wenzhou, Zhejiang Province, China; 3 School of Basic Medical Sciences, Shandong Medical College, Linyi, Shandong Province, China; 4 School of Economics, Huazhong University of Science and Technology, Wuhan, Hubei Province, China; Max Delbruck Centrum fur Molekulare Medizin Berlin Buch, GERMANY

## Abstract

Resveratrol (RSV), a natural polyphenol, has been reported to produce effect on genes transcription in lipid metabolism. In this study, we aim to explore the novel mechanisms of RSV on the regulation of caveolin-1 (Cav-1) transcription. Via body weight, blood glucose, serum lipid, and liver pathology detection, we found that RSV decreased body weight, blood glucose and lipid accumulation in rats fed high-fat diet. Based on co-immunoprecipitation (Co-IP) and western blotting assay, we found that RSV up-regulated klf5 phosphorylation and decreased the interaction of klf5 with c-Myc, which were accompanied by down-regulation of Cav-1 expression in livers of rats fed with high-fat diet. Moreover, in HEK293 cells, we observed RSV enhanced klf5 phosphorylation and separated the interaction of klf5 with c-Myc through inhibiting the activation of PI3K/PKD1/Akt pathway, which maybe promoted c-Myc binding to the promoter to inhibit Cav-1 expression. The results of the present study demonstrated that RSV activated klf5 phosphorylation by inhibiting PI3K/PKD1/Akt pathway, and then attenuated the interaction of klf5 with c-Myc, subsequently probably promoted the c-Myc binding to the promoter to repress Cav-1 expression.

## Introduction

Caveolae, plasma membrane invaginations, plays a specific role in maintaining cellular cholesterol transportation and energy balance. Caveolin-1 (Cav-1) is an integral membrane protein and the main structural protein of caveolae in nonmuscle cells. It has been reported that Cav-1 is also important in the maintenance of hepatic lipid homeostasis[1]. In fact, previous studies have extensively demonstrated that Cav-1 and caveolae contribute to regulate the hepatic energy metabolism by fatty acid oxidation [1,2]. By contrast, transcriptional regulation of Cav-1 is still only poorly understood, though the transcription factor c-Myc was known to be involved [3].

Krüppel-like factor 5 (klf5) as a zinc-finger transcription factor regulated various biological processes, including adipocyte differentiation programs through its interaction with C/EBP-β and C/EBP-δ and through transactivation of Pparg2, encoding PPAR-gamma2[4], and transcriptional programs of lipid metabolism involving PPAR-δ by klf5-SUMOylated in skeletal muscle[5]. Earlier studies have identified klf5 directly interacted with c-Myc in a TGF-β-dependent manner to regulate cell proliferation [6]. Furthermore, several studies have also demonstrated the ability of Myc to trans-activate or -repress a repertoire of genes is the main mechanism for its ability to mediate cellular metabolic switch [7,8]. However, the role of klf5 and c-Myc in the regulation of lipid metabolism in adult animals has not yet been addressed.

Resveratrol (3, 4', 5-trihydroxystilbene, RSV), a natural polyphenol, has been identified to be beneficial for obesity and obesity-associated diseases such as NAFLD in vitro and in vivo [9]. Recent study has indicated RSV ameliorates hepatic fibrosis and inflammation in a mouse model of nonalcoholic steatohepatitis [10]. Additionally, class IA phosphoinositide 3-kinases (PI3Ks) was found to be involved in HFD-induced liver steatosis [11]. PI3Ks consisted of catalytic subunit (p110) and regulatory subunits (collectively called p85) are recruited to the plasma membrane in response to growth factor and hormone stimulation to mediate the phosphorylation of lipid phosphatidylinositol-4,5-bisphosphate (PIP2), generating phosphatidylinositol-3,4,5-trisphosphate (PIP3) [12]. PIP3 bonded to the pleckstrin homology domain of Akt and membrane translocation of another target of PIP3, PDK1, which phosphorylates and activates Akt [13]. Akt plays a fundamental role in energy metabolism [13].

In the present study, we demonstrated that RSV inhibited Cav-1 expression through activating klf5 phosphorylation for separating the interaction of klf5 with c-Myc in livers of rats fed high-fat diet. Moreover, PI3K/PKD1/Akt pathway was repressed after RSV treatment, which promoted klf5 phosphorylation, subsequently leading to the inhibition of the interaction of klf5 with c-Myc and the transactivation function.

## Materials and methods

### Reagents and antibodies

Resveratrol (purity > 99%) was obtained from Nanjing Spring & Autumn Biological Engineering Co., Ltd. (Nanjing, China). PI3Kp85, phospho-PI3Kp85 (Tyr458), Akt, phospho-Akt (Ser473), phospho-PKD1 (Ser916), c-Myc, and Cav-1 antibodies were purchased from Cell Signaling Technology Inc. (Beverly, MA, USA). PKD1 was from Novus (Novus Biologicals, Littleton, Colorado). Klf5 antibody was obtained from Abcam (Cambridge, MA, USA). The phosphoserine antibody was purchased by Santa Cruz Biotechnology Inc. (Santa Cruz, USA). GAPDH antibodies were purchased from proteintech (Wuhan, China). Trizol was from Invitrogen Inc. (Carlsbad, CA, USA). Reverse transcription reaction Kit was from Toyobo Co.Ltd. (Osaka, Japan) and quantitative Real-time PCR kit was purchased from TAKARA Bio Inc. (Otsu, Shiga, Japan). All other chemicals were of the highest grade commercially available.

### Animal experiments

Thirty adult male Wistar rats (about 40–60 g) were obtained from Shanghai SLAC Laboratory Animal Co., Ltd. (Shanghai, China). All of the rats, after one weeks' acclimation, were randomly divided into three groups: a standard diet (Con, n = 10), a high-fat diet (HFD, n = 10), and a HFD+RSV (100 mg/kg/day, n = 10). The high-fat diet included 53% fat, 32.5% carbohydrate and 14.5% protein, while the normal standard diet consisted of 2.85% fat, 77.15% carbohydrate and 20% protein. At the end of 18th week, all animals were euthanized. Blood was collected. The liver tissues were weighed, and fixed in neutral buffered 4% paraformaldehyde (PFA) or snap frozen with liquid nitrogen then stored at -80°C freezer until further use. The animal experiments were approved by the Committee on the Ethics of Animal Experiments of the Hua zhong University of Science and Technology (Permit number: S249).

### HEK293 cell culture

Human embryonic kidney 293 (HEK293) cell line was obtained from Shanghai Cell Bank of Chinese Academy of Sciences (Shanghai, China). HEK293 were cultured in Dulbecco’s modified Eagle’s medium (DMEM) supplemented with 10% fetal bovine serum (FBS) and 1% penicillin/streptomycin. Cells were treated with or without RSV (20 μM) for different time intervals (12, 24 and 48 h). Cultured HEK293 in DMEM high glucose (25 mmol/L, HG) were treated without RSV, treated with inhibitor LY294002 for 2 h, or with RSV (20 μM) for 24 h, respectively [14,15]. HEK293 incubated with 5.5 mmol/L glucose in medium were used as control[15].

### Blood biochemical indexes

After 18th week, after an overnight fasting, the blood of the rats was collected via tail vein and centrifuged for the measurement of blood glucose and serum lipid parameters. The total cholesterol (TC), triglyceride (TG), low-density lipoprotein cholesterol (LDL-C) and high density lipoprotein (HDL) were measured according to the manufacturer’s instruction.

### Liver weight and liver index

At the end of the experiment, livers of all rats were removed and weighed. The liver index were evaluated by the ratio of liver weight and body weight.

### Hematoxylin and eosin staining

The livers were fixed in neutral buffered 4% paraformaldehyde (PFA). At room temperature, the samples were embedded in paraffin and sectioned to 4-μm-thick by pathologic microtome (RM2016, Leica), and then fixed to glass slides and dried at 60°C. At last, the tissue sections were deparaffinised and stained with hematoxylin and eosin (H&E) or oil red O staining, and observed at 200× magnifications by optical microscope (Nikon Eclipse CI, Japan).

### Immunoblotting

Co-immunoprecipitation (Co-IP) was performed according with previously described [6]. Total protein was extracted from the livers by RIPA Lysing Buffer. The protein concentration was quantified with BIO-RAD Dc protein assay reagent (Bio-Rad, Hercules, USA) in accordance with the manufacturer's instruction. Equal amounts of protein was used to sodium dodecyl sulfate polyacrylamide gel electrophoresis (SDS-PAGE) and immunological blotting (IB). Protein expression was visualized with a chemiluminescent detection system (Syngen, Cambridge, UK) and analyzed by GelPro3.0 software (Biometra, Goettingen, Germany).

### Quantitative real-time PCR

Total RNA was extracted from isolated liver tissues and then quantified using UV spectrophotometry. Samples were accepted in according with the A260/280 ratio lies between 1.8 and 2.0. For all the samples, 1 μg total RNA were used to synthesize cDNA according to the manufacturer’s instruction. Real time PCR analysis was carried out using qPCR SYBR Green mix on an ABI PRISM 7900 machine (Applied Biosystems) under the following conditons: 1 cycle, 95°C, 5s; 40 cycles, 95°C, 10s; 57°C, 30s. Relative gene expression were determined by using the 2^-ΔΔCt^ method, and GAPDH was used as endogenous reference gene. The primers used in the real-time PCR were as follows: (1)rats: Cav-1 primer was 5′-TCTACAAGCCCAACAACAAGGCC-3′ (sense) and 5′-TGCACTGAATCTCAATCAGGAAGC-3′ (antisense), TGF-β primer was 5′-AACCAAACTCACGGATGAGG-3′ (sense) and 5′-TTCCCGTCAGTCTTGCTTCT-3′ (antisense), GAPDH primer was 5′-GACAACTTTGGCATCGTGGA-3′ (sense) and 5′-GGTCTTGTAGTAGGGACGTA-3′ (antisense); and (2) Human: Cav-1 primer was 5′-GCGACCCTAAACACCTCAAC-3′ (sense) and 5′-ATGCCGTCAAAACTGTGTGTC-3′ (antisense), GAPDH primer was 5′-TGTGGGCATCAATGGATTTGG-3′ (sense) and 5′-ACACCATGTATTCCGGGTCAAT-3′ (antisense).

### Statistical analysis

All data were presented as means ± SD (Standard Deviation). Data processing and statistical analysis were performed with SPSS 20.0 (SPSS Inc. Chicago, IL, USA). Data were compared by one-way analysis of variance (ANOVA) or Dunnett’s T3 test. Differences were considered significant when a value of *P* < 0.05.

## Results

### Effects of RSV on body weight, blood glucose and serum lipid levels in HFD fed rats

Data are means ± SD (n = 6). Differences were considered significant when a value of *P* < 0.05.

Rats fed HFD exhibited a significant increase in body weight compared with the rats in SD group. Whereas, rats in HFD+RSV group showed a significant decrease in body weight compared to the rats in HFD group (Table 1). In addition, blood glucose levels was significantly increased by HFD feeding, but RSV treatment did not affect the blood glucose levels (Table 1). In the plasma biochemical analysis, TC, TG, LDL-C, and HDL-C levels in the HFD group were significantly higher than in SD group, but significant difference was observed between HFD group and HFD with RSV group. The increased TC, TG, LDL-C, and HDL-C levels in rats fed HFD were decreased by RSV (Table 1).

**Table 1:** Effects of resveratrol on body weight, blood glucose and serum lipid.

Parameters	SD	HFD	HFD+RSV	*P* value	
				HFD vs. SD	HFD+RSV vs. HFD
Initial body weight (g)	192.56± 9.09	196.44± 9.28	195.60±12.58	0.68	0.999
Final body weight (g)	505.75±22.25	618.89±18.24	545.00±21.62	<0.001	<0.001
Blood glucose (mmol/L)	6.34±0.61	6.87±0.49	6.95±0.69	0.037	0.735
TC (mmol/L)	1.63±0.16	2.00±0.26	1.47±0.18	0.007	<0.001
TG (mmol/L)	0.78±0.16	1.27±0.20	0.72±0.14	0.004	<0.001
LDL-C (mmol/L)	0.29±0.12	0.68±0.11	0.21±0.10	<0.001	<0.001
HDL-C (mmol/L)	1.34±0.28	0.84±0.26	1.39±0.15	0.002	<0.001

### Ameliorative effects of RSV on liver damage induced by HFD

**Fig 1:**
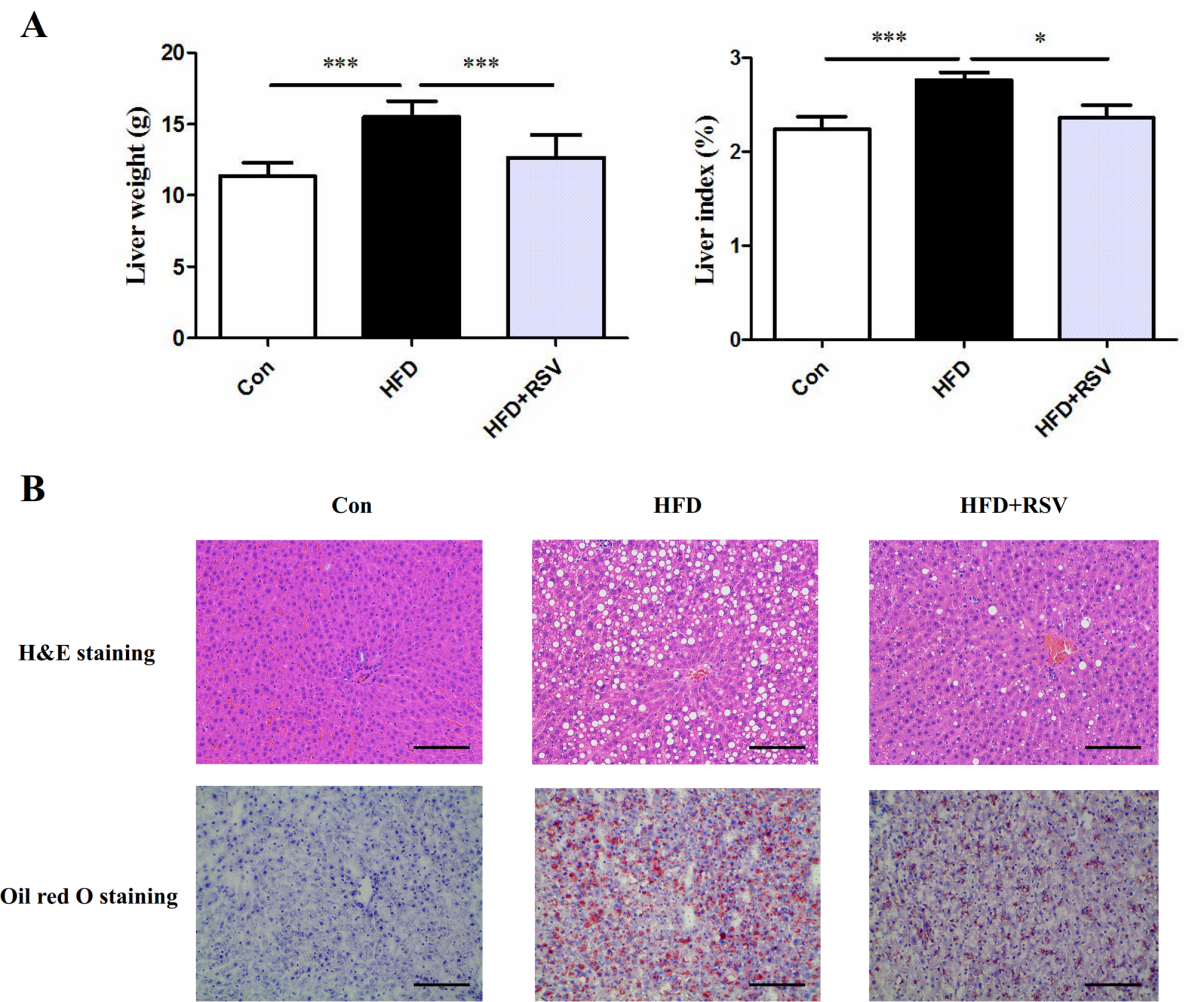
Effects of RSV on liver weight, liver index and histological changes in HFD rats. (A) High-fat diet increased the liver weight and liver index, while RSV treatment significantly decreased the liver weight and liver index gain compared with HFD group. (B) The liver histological detection indicated large hepatic lipid droplets in rats fed HFD, while RSV treatment significantly ameliorated the effects (magnification: ×200). Data was presented as means ± SD (n = 10), (***P < 0.001, *P < 0.05).

The liver weight and liver index were markedly higher in HFD group than in SD group (Fig 1A). RSV treatment showed a significant decrease on the liver weight and liver indexes in rats fed HFD (*P* < 0.001 and *P* < 0.05, respectively). Meanwhile, to test the protective effects of RSV on hepatic histology, we performed the H&E staining and oil red O staining of liver section and found the liver in SD group had normal histology, while in HFD group, the liver was characterized by a large number of fat depositions (Fig 1B). However, RSV treatment significantly reduced the numbers of fat droplets of liver in HFD fed rats (Fig 1B).

### RSV enhanced klf5 phosphorylation, and inhibited the interaction between klf5 and c-Myc in livers of rats fed HFD

**Fig 2:**
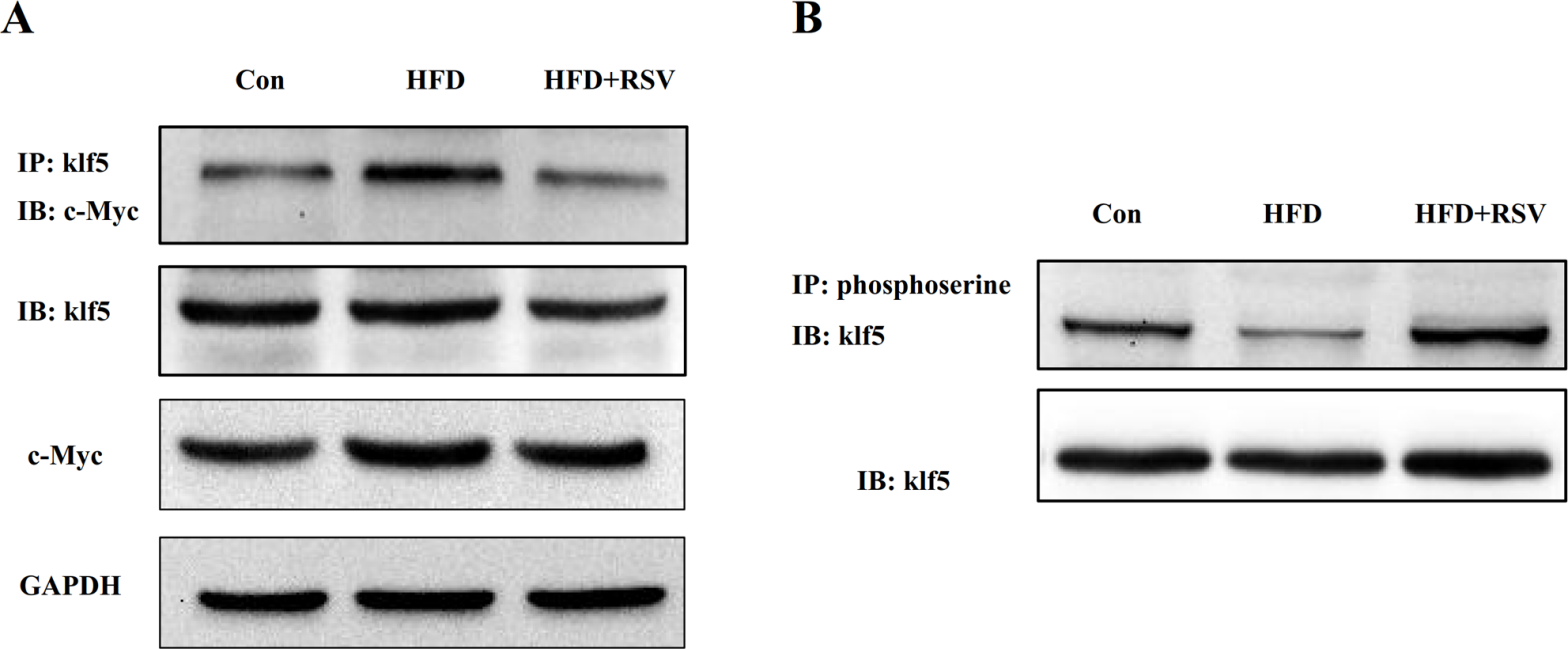
RSV attenuated the interaction between klf5 and c-Myc and increased the klf5 phosphorylation in livers of rats fed high-fat diet. The total protein was extracted from livers of rats. (A) The extracts were subjected to immunoprecipitation with anti-klf5 antibody as indicated (IP). The precipitates were analyzed by western blotting analysis. (B) The extracts were immunoprecipitated with antibody to phosphoserine and immunoblotted with antibody to klf5.

To understand if RSV could affect the interaction between klf5 and c-Myc, we performed a co-immunoprecipitation (Co-IP) assay by using crossing Co-IP. We found that the RSV-administered group showed significantly less interaction between klf5 and c-Myc than model groups (Fig 2A). Apparently, RSV inhibits the association of klf5 with c-Myc in the liver tissues. Moreover, we tested the klf5 phosphorylation level as well, the result demonstrated that high-fat diet decreased the klf5 phosphorylation level, whereas RSV could reverse this effect (Fig 2B). Importantly, the total klf5 and c-Myc protein levels were unchanged by high-fat diet or RSV (Fig 2A). These results indicated that RSV played a vital role on klf5 phosphorylation level, and probable influence its transactivation function.

### RSV suppressed the activation of PI3K/PKD1/Akt pathway in livers of rats fed HFD

**Fig 3:**
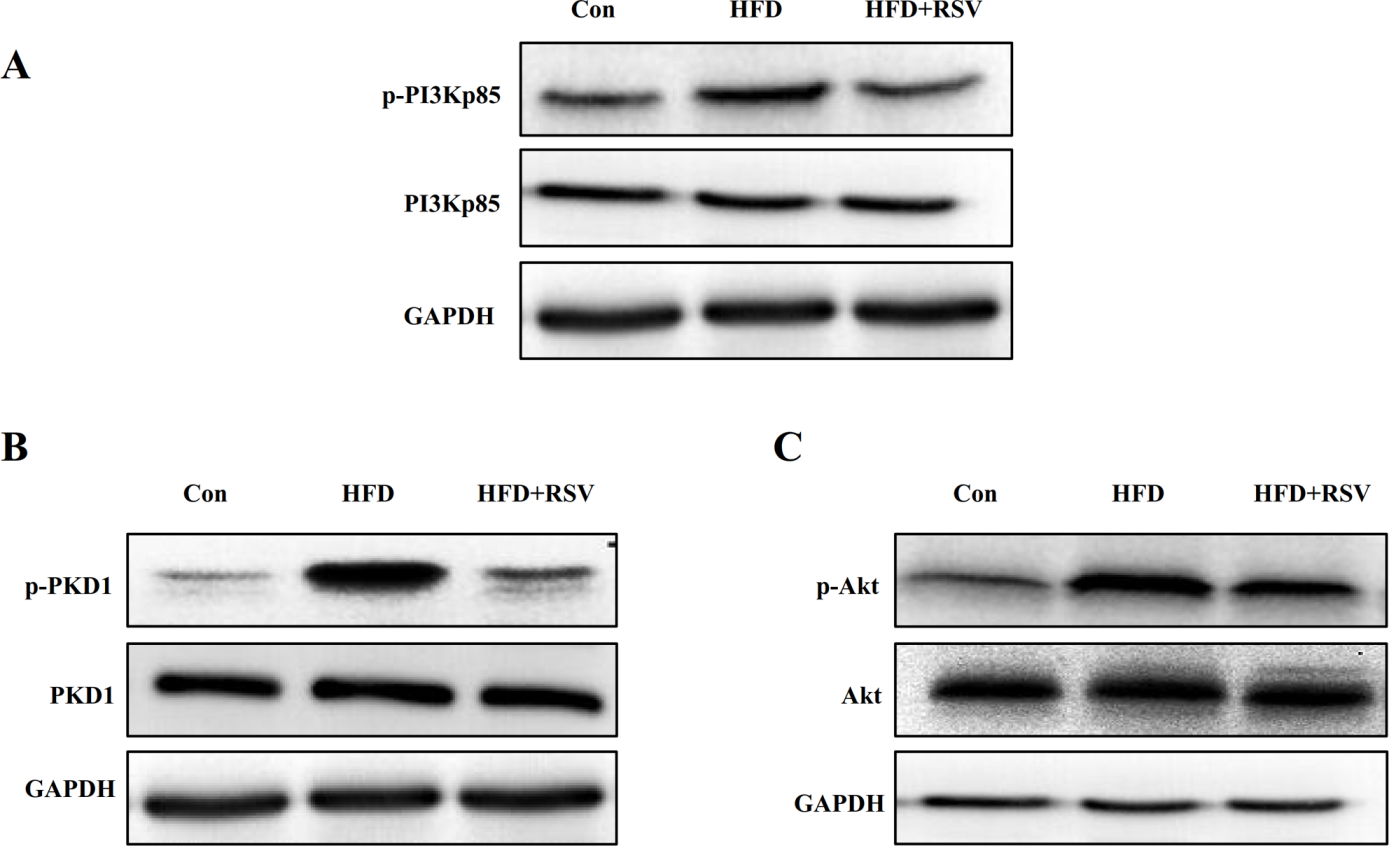
RSV inhibited the activation of PI3K/PKD1/Akt pathway induced by HFD in livers of rats. The total protein was extracted from livers of rats. Western blotting was performed on cell lysates using phospho-specific antibodies to detect phosphorylated PI3Kp85 (A), phosphorylated PKD1 (B), and phosphorylated Akt (C), respectively. Blots for total protein were also shown (PI3K, PKD1 and Akt).

It is known that activation of Akt signaling is crucial for many fundamental cellular processes. To determine the activation of PI3K/PKD1/Akt pathway, we examined the levels of phospho-PI3K, phospho-PKD1, and phospho-Akt by western blotting using phospho-specific antibodies. Fig 3A–3C shows that p-PI3K, p-PKD1, and p-Akt remarkably increase in HFD group compared to control group and HFD+RSV group, however, RSV supplement in high-fat diet can decrease PI3K, PKD1, Akt phosphorylation level compared with HFD group.

### RSV inhibits the up-regulation of TGF-β and caveolin-1 expression induced by HFD

**Fig 4:**
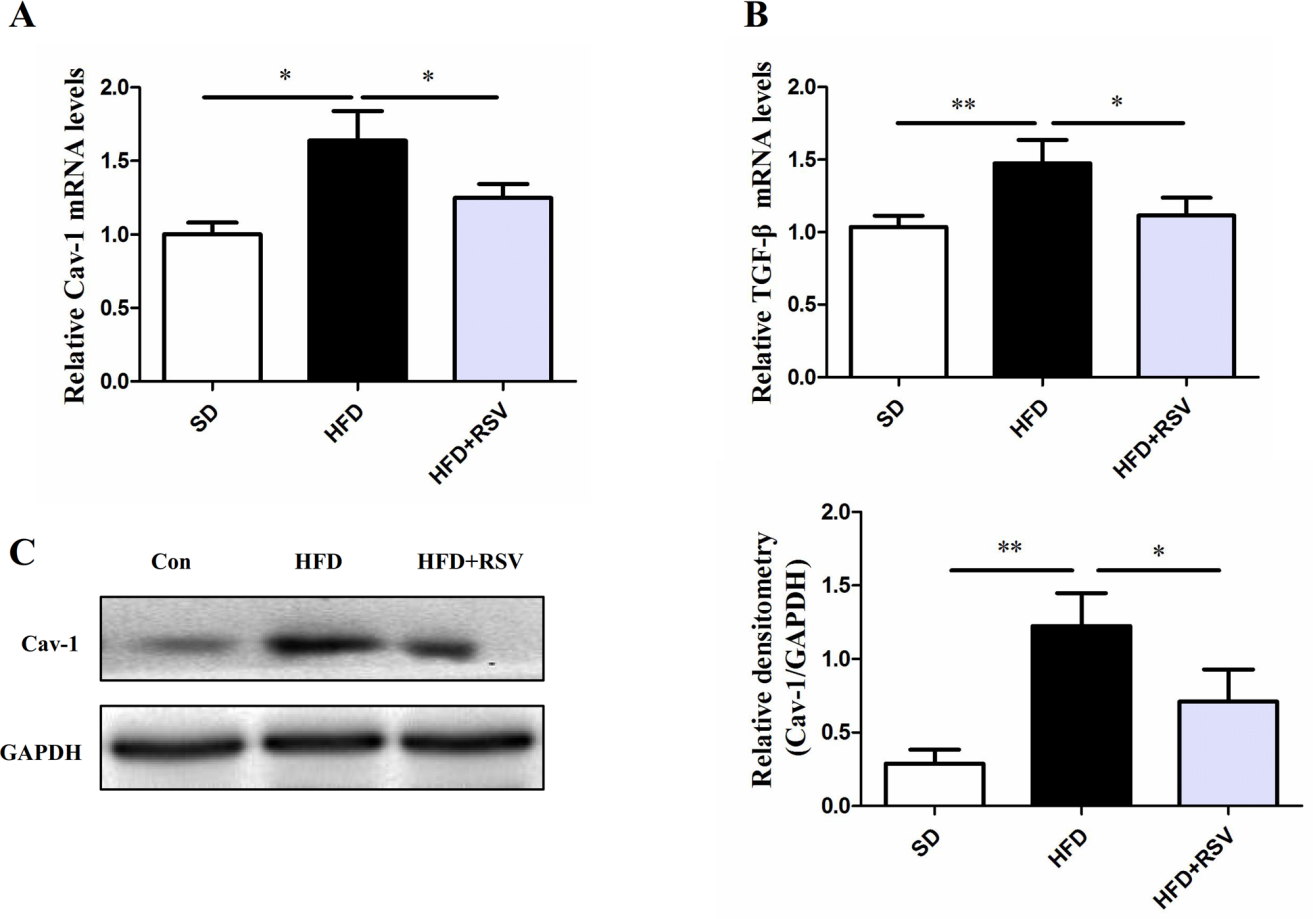
RSV decreased the Cav-1 and TGF-β expression in liver of rats fed high-fat diet. Protein levels was calculated with GADPH as reference. High-fat diet increased the levels of Cav-1 mRNA (A) and protein (C), and level of TGF-β mRNA (B), while RSV treatment significantly decreased Cav-1 and TGF-β expression in HFD rats. Data was presented as means ± SD; (**P < 0.01 and *P < 0.05).

Caveolae and Cav-1 play important roles in energy balance and cholesterol transport. To examine the effect of high-fat diet and interaction of klf5 with c-Myc on Cav-1 expression, we tested the mRNA and protein levels of Cav-1. As shown in Fig 4A and 4C, high-fat diet up-regulated the expression levels of Cav-1 mRNA and protein in fed high-fat diet rats. RSV supplement in high-fat diet significantly down-regulated its mRNA and protein levels compared with HFD group. In addition, HFD up-regulated the TGF-β mRNA level, while RSV could reverse the effect (Fig 4B).

### RSV inhibits the interaction between klf5 and c-Myc by increasing klf5phosphorylation level in HEK293 cells

**Fig 5:**
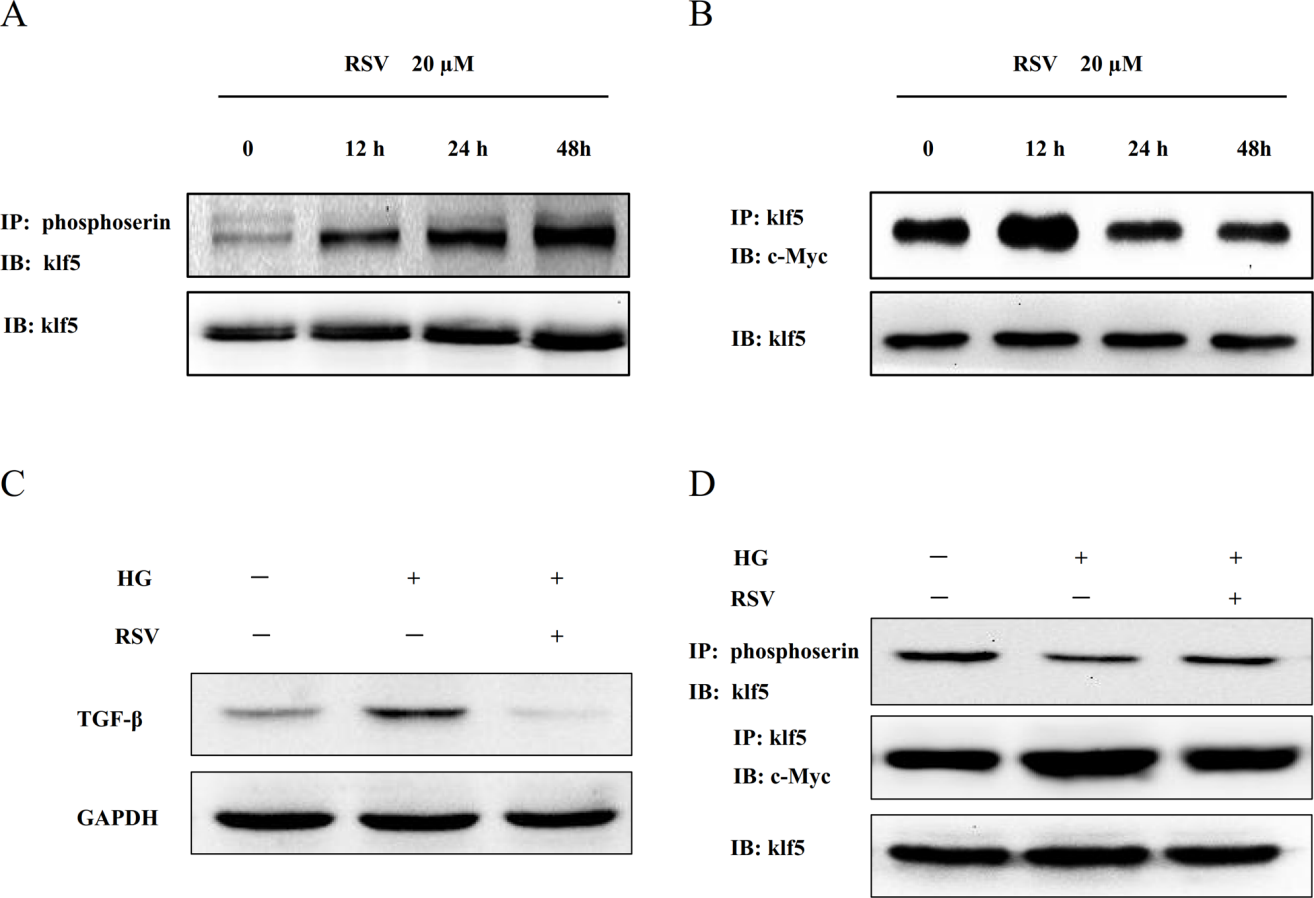
RSV inhibited the interaction of klf5 with c-Myc by inducing klf5 phosphorylation. Cultured HEK293 cells were incubated with 20 μM RSV for different times, and then subjected to western blot analysis. (A) Cell extracts were immunoprecipitated with antibody to phosphoserine and immunoblotted with antibody to klf5. (B) Cell extracts were immunoprecipitated with antibody to klf5 and immunoblotted with antibody to c-Myc. (C) RSV treatment significantly decreased TGF-β expression in HEK293 cells. (D) RSV increased the klf5 phosphorylation, and promoted the the interaction of klf5 with c-Myc.

As shown in Fig 5, RSV induced phosphorylation of klf5 in a time-dependent manner, while RSV treatment reduced the interaction of klf5 with c-Myc in 24 h and 48h (Fig 5A and 5B). However, HEK293 cells detached from the culture plate when cell was incubated for 48 h. Additionally, RSV down-regulated the TGF-β protein level, increased the klf5 phosphorylation, and promoted the the interaction of klf5 with c-Myc (Fig 5C and 5D).

### Inhibitor LY294002 and RSV prevented the interaction of klf5 with c-Myc and increased klf5 phosphorylation level via PI3K/PKD1/Akt pathway in HEK293 cells cultured in EMDM with high glucose

**Fig 6:**
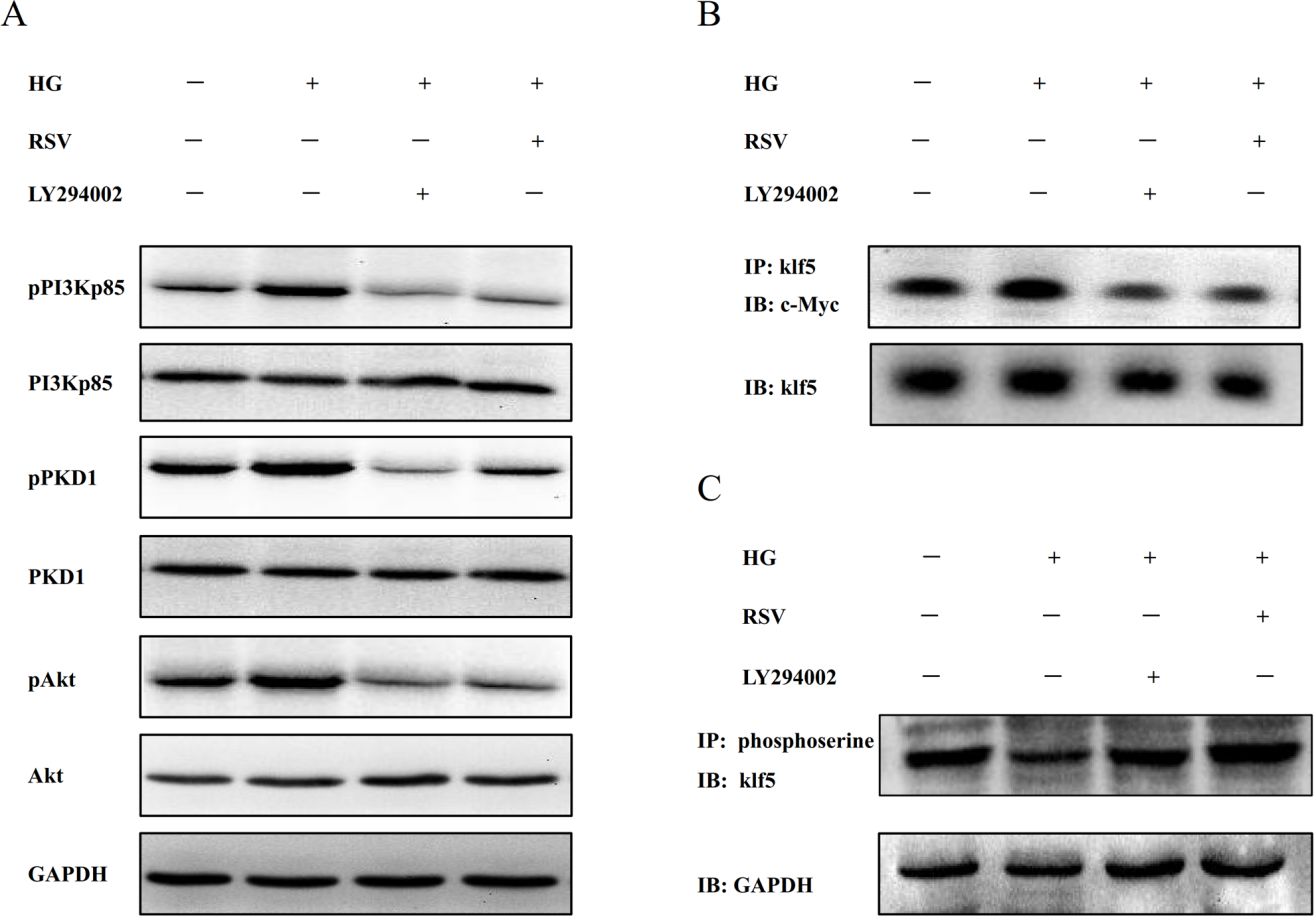
Inhibitor LY294002 and RSV attenuated the interaction of klf5 with c-Myc and up-regulated klf5 phosphorylation via PI3K/PKD1/Akt pathway in HEK293 cells cultured in DMEM high glucose. The cultured HEK293 cells were treated with RSV and LY294002, respectively. High glucose incubation up-regulated the interaction of klf5 with c-Myc (B), phosphorylation of PI3K, phosphorylation of PKD1 and phosphorylation of Akt (A), and decreased klf5 phosphorylation (C). All the above effects could be reversed by RSV or LY294002.

To explore whether activated PI3K/PKD1/Akt pathway is involved in HG-induced klf5 dephosphorylation and the promoted the interaction between klf5 and c-Myc, we tested the effects of PI3K inhibitor LY294002 on HG-induced klf5 dephosphorylation and the interaction between klf5 and c-Myc in HEK293 cells cultured in DMEM high glucose. As shown in Fig 6A–6C, LY294002 and RSV inhibited the activation of PI3K/PKD1/Akt pathway, repressed the interaction of klf5 with c-Myc, and increased klf5 phosphorylation. These results suggested that PI3K/PKD1/Akt pathway is involved in the interaction of klf5 with c-Myc and klf5 dephosphorylation in HEK293.

### Inhibitor LY294002 and RSV block the Cav-1 expression in HEK293 cells cultured in DMEM with high glucose

**Fig 7:**
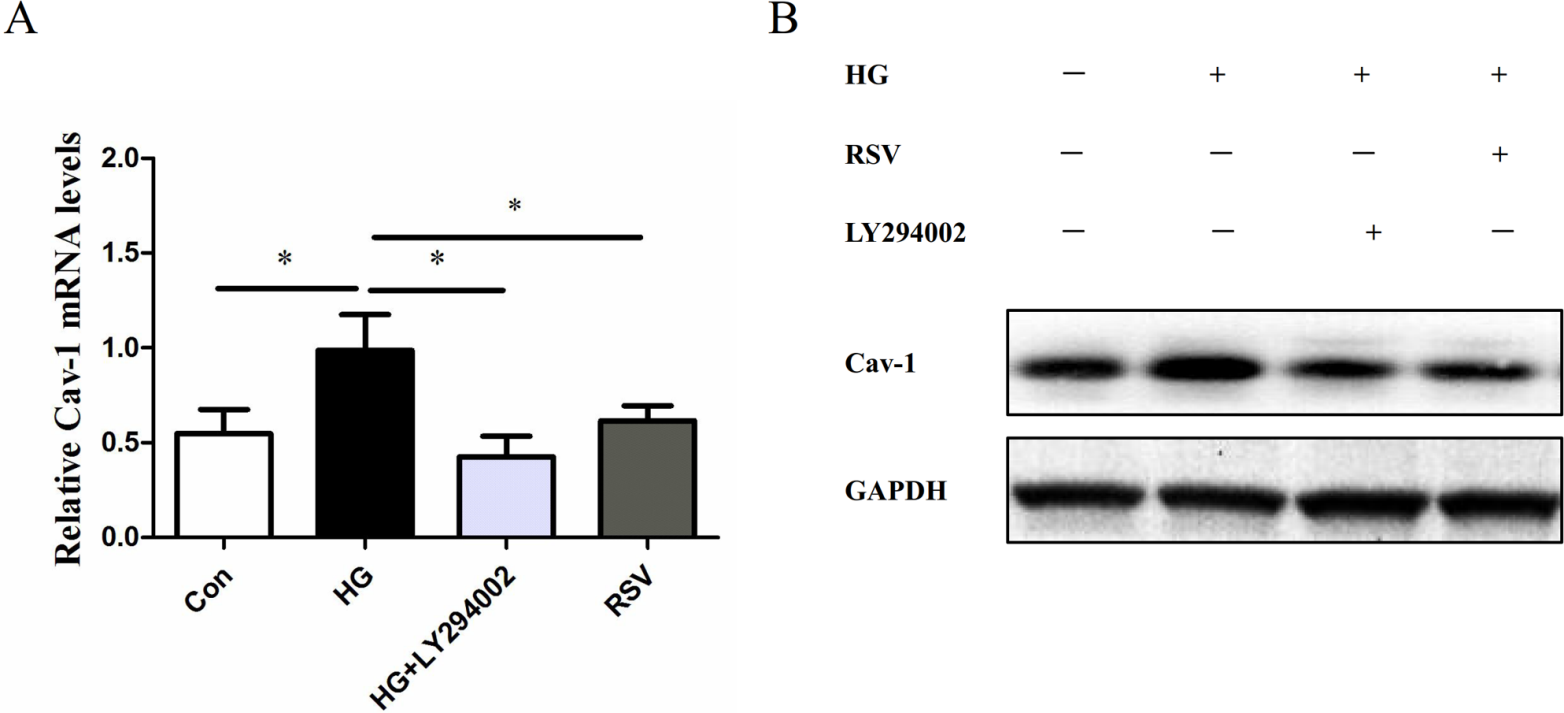
Inhibitor LY294002 and RSV block the Cav-1 expression in HEK293 cells cultured in DMEM with high glucose. The cultured HEK293 cells were treated with RSV and LY294002, respectively. High glucose incubation up-regulated the Cav-1 mRNA and protein levels, while RSV or LY294002 could down-regulated the Cav-1 mRNA and protein levels. Data was presented as means ± SD, (*P < 0.05).

As shown in Fig 7, HG up-regulated the expression of Cav-1 mRNA and protein levels. Inhibitor LY294002 and RSV block the up-regulation of Cav-1 mRNA and protein levels induced by HG in HEK293 cells.

## Discussion

With the changes of dietary ingredient and physical activity patterns in our modern world, obesity has rapidly increased in the last few decades, especially in Europe as well as in the UK. The increasing prevalence of obesity has increased the epidemic of non-alcoholic fatty liver disease (NAFLD) around the world. The present study confirmed that rats received a high-fat diet provoked an obvious obesity characterized by increased body weight gain, pathoglycemia, dyslipidemia. We also found that high-fat diet increased liver weight and liver index and promoted overt hepatic histological changes in rats. Previous studies have shown that RSV exerted the potential anti-obesity effect, alleviated hepatic histological changes, and inhibited nonalcoholic fatty liver disease in mice[16,17]. Similarly, we observed that oral supplementation of RSV at dose of 100mg/kg of body weight markedly decreased body weight and redressed pathoglycemia and dyslipidemia in high-fat fed rats. Additionally, RSV treatment reduced liver weight and liver index as well as attenuated hepatic steatosis induced by the high-fat diet. These results indicated that RSV played an important role in preventing obesity and ameliorating liver damage in rats fed HFD.

Caveolae and Cav-1 have been demonstrated to be necessary for hepatic oxidative lipid metabolism high-fat feeding independently of the genetic background [1]. Cav-1 maintained the balance between glucose and lipid-dependent energy metabolism, whcih have been implicated in liver regeneration [3]. Furthermore, Cav-1 deficiency or overexpression of Cav-1 (mutants) as well as dysregulation of caveolar function have been closely related to disease pathogenesis of liver cancer, metabolic adaptation, liver regeneration and lipotoxicity [18]. Therefore, it is urgent to study how the Cav-1 gene expression is regulated in the process of intracellular and organism activities. In our study, we found that rats received high-fat diet showed high expression of Cav-1 mRNA and protein levels in liver of rats, however, Cav-1 expression was significantly depressed when RSV was applied. From previous study showed c-Myc could directly bind to the promoter to repress Cav-1 gene expression via an INR-dependent mechanism [3], and c-Myc directly interacted with klf5 only when transforming growth factor beta (TGF-β) was activated [6]. Interestingly, RSV could inhibit TGF-β1 and TGF-β2 gene expression and activation[19,20], which were consistent with our studies. Therefore, we performed a co-immunoprecipitation (Co-IP) assay for evaluating the interaction between klf5 and c-Myc, the results showed HFD induced the interaction of klf5 with c-Myc, but RSV significantly repressed the effect. Furthermore, we detected the levels of klf5 phosphorylation, suggesting klf5 phosphorylation level in HFD group was lower than HFD+RSV group. In parallel, in HEK293 cells, we also observed that HG (25 mmol/L) enhanced the interaction of klf5 with c-Myc and klf5 dephosphorylation, which were reversed by treatment with inhibitor LY294002 and RSV. Given these findings, we hypothesized that klf5 phosphorylation maybe involve in the interaction of klf5 with c-Myc.

To investigate whether the change in klf5 phosphorylation is responsible for RSV-induced suppression of interaction between klf5 and c-Myc, we detected the levels of phospho-klf5 in HEK293 cells treated with RSV. The result showed that RSV enhanced the klf5 phosphorylation which has a negative correlation with the interaction between klf5 and c-Myc in a time-dependent manner. Therefore, phosphorylation of klf5 is vital for the interaction between these two proteins.

Protein phosphorylation has been an important mechanism by which a transcription activator recruits coactivators, such as phosphorylation of klf5 at the cAMP response element-binding protein (CREB)-binding protein (CBP) interaction region enhancing its interaction with CBP and its transactivation function, phosphorylation of CREB at Ser133 leading to the recruitment of CBP [21,22]. Previous study has also demonstrated klf5-dephosphorylated inhibited interaction of klf5 with RARa through inducing klf5 dephosphorylation directly mediated by the PI3K/Akt signaling in vascular smooth muscle cells [23]. Furthermore, Several studies have also demonstrated that Klf5 interacted with cofactors and modified (e.g., acetylated and phosphorylated) to regulate klf5 transcriptional function. For example, klf5 activity is increased through its interaction with the coactivator/acetylase p300 [24], and deacetylase (histone deacetylase 1 [HDAC1]) negatively regulates the transcriptional activity of klf5 by direct interaction as well as inhibition of its interaction with p300 [25], and phosphorylation of klf5 at the CBP interaction region enhances its activation and its transactivation function[21]. Thus, we examine the effect of RSV regulates the klf5 phosphorylation. We observed that HFD increases klf5 dephosphorylation, however, RSV could attenuate the effect. To understand the mechanisms of RSV repression the klf5 dephosphorylation, we then examined the activation of PI3K/PKD1/Akt pathway in HEK293 treated with HG, HG with inhibitors LY294002, and HG with RSV (20μM). These results showed that LY294002 and RSV, under HG condition, could significantly inhibit the klf5 dephosphorylation via decreasing activation of PI3K/PKD1/Akt pathway in dephosphorylation way. Thus, RSV represses the Cav-1 expression via inhibiting the activation of PI3K/PKD1/Akt pathway, and then blocking the klf5 dephosphorylation and the interaction of klf5 with c-Myc, subsequently c-Myc direct binding to the promoter and preventing the Cav-1 expression.

## Conclusions

In summary, we have found that RSV repressed klf5 dephosphorylation, subsequently contributing to separating the interaction of klf5 with c-Myc, and promoting the inhibition of Cav-1 expression. Furthermore, RSV attenuated the klf5 dephosphorylation via PI3K/PKD1/Akt pathway. More importantly, our study maybe provide not only novel insight into the molecular mechanism of the inhibitory effect by RSV but also a specific therapeutic target for NAFLD induced by high-fat diet.
